# First person – Favour Ononiwu

**DOI:** 10.1242/bio.062530

**Published:** 2026-03-24

## Abstract

First Person is a series of interviews with the first authors of a selection of papers published in Biology Open, helping researchers promote themselves alongside their papers. Favour Ononiwu is first author on ‘ Insights into the zebrafish left–right organizer's centrosomes and cilia via volume electron microscopy’, published in BiO. Favour conducted the research described in this article while a PhD graduate student in Dr Heidi Hehnly's lab at Syracuse University, Syracuse, NY, USA. She is now a postdoc in the lab of Dr Amy Kiger at University of California San Diego (UCSD), La Jolla, CA, USA, investigating mechanisms that drive tissue-scale morphogenesis, and maintenance in developmental and disease settings.


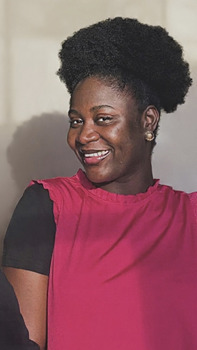


**Favour Ononiwu**

**Describe your scientific journey and your current research focus**

Growing up in Nigeria, cassava was a staple, and my earliest memory of curiosity involved helping my grandmother ferment it, observing its transformation. Years later, I remembered this experience during senior secondary school when learning about fermentation and microorganisms. Around the same time, hospital visits with my nephrologist mother exposed me to the clinical world, fuelling my interest in biological systems and their application to human health. These experiences, alongside the realization that scientific knowledge is a human endeavour rather than mystical, led me to pursue an undergraduate degree in Microbiology and Industrial Biotechnology. My initial research experience solidified that textbook information was grounded in real-world application, and, during graduate studies, I cultivated expertise in tissue culture. Dr Allison Oakes later trusted me to draft and teach a 2- to 3-week science lab focused on tissue culture, reportedly one of the department's first hands-on animal tissue culture courses. This experience allowed me not only to grow as a scientist but also to contribute to the educational programming of younger scientists. My doctoral training under Dr Heidi Hehnly was transformative, equipping me with the technical foundation and confidence to investigate cellular processes with greater depth and independence. Now, as a postdoc in Dr Amy Kiger's lab at UCSD, I see how all these distinct experiences and the invaluable contributions of these individuals are interconnected and have now shaped my scientific journey, as well as my current research interests focused on elucidating how cellular events drive both the formation maintenance of tissues. Using the fruit fly as a model organism, my current research explores how muscles are maintained through regulated remodelling, focusing on the cellular mechanisms that drive its integrity, and I specifically investigate two critical membrane components, T-tubules and costameres, to uncover the regulatory mechanisms governing their coordination.

**Who or what inspired you to become a scientist?**

My inspiration to become a scientist came from Mr Olatope Olakitan, an industry research scientist who provided unofficial mentorship during my undergraduate years, and my elder brother, an organic chemist whose passion and work ethic offered a strong example.

**How would you explain the main finding of your paper?**

Our bodies usually place organs in very specific positions; for example, the heart forms on the left side and the liver on the right. This arrangement depends on a short-lived structure that appears very early in development. Inside this temporary structure are tiny, hair-like projections that stir fluid in a particular direction. That movement helps send signals that tell the body where each organ should develop. In our study, we examined this structure in zebrafish in exceptionally fine three-dimensional (3D) detail.

What we found was unexpected. The tiny hair-like projections are not all built the same way, and the structures at their base, which anchor and organize them inside the cell, also vary from cell to cell. Some have complete structural components, others appear partially remodelled, and a small number lack elements that were previously assumed to be universally present. In other words, this early developmental structure is more diverse and less uniform than scientists once thought. Rather than being a collection of identical parts performing the same task, it appears to be made up of cells with subtly different structural features. This hidden diversity suggests that the system guiding left–right organ placement may be more complex and adaptable than previously appreciated.

**What are the potential implications of this finding for your field of research?**

These findings open new avenues for researchers to explore the specific implications of various structural compositions of this hair-like structure known as the cilia and how associated structures contribute to ciliary beating, especially in the context of generating directional flow. By understanding these intricate details, the cilia field can potentially identify mechanisms that, if disrupted, could lead to congenital defects in organ laterality. This deeper understanding of their structural diversity gives us new clues about how the body achieves such precise left–right organization.

Spotting the Kupffer's vesicle within the entire zebrafish embryo without a marker was a major triumph

**Figure:**
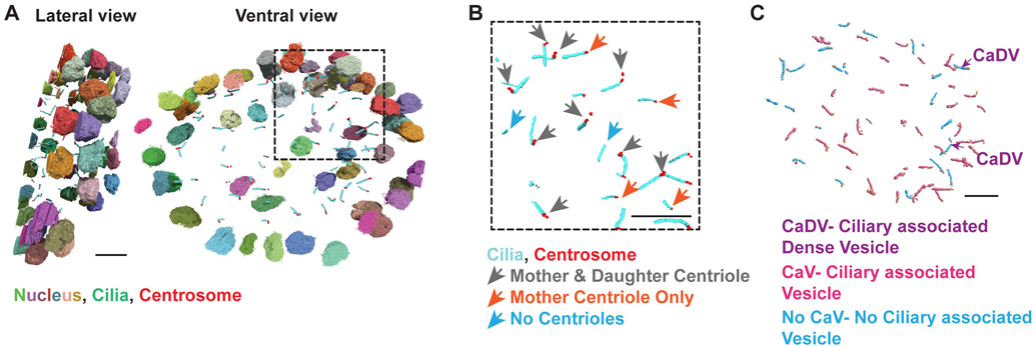
**3D reconstruction of segmented zebrafish left–right organizer (LRO) showing structural heterogeneity in subcellular structures – cilia and centrosome.** (A) 3D volume electron microscopy reconstruction of the zebrafish LRO, showing lateral (left) and ventral (right) views of the segmented volume: nuclei (multi-coloured), cilia (cyan) and centrosomes/centrioles (orange). (B) Zoomed inset shows centrosome heterogeneity and ciliary heterogeneity: cilia with both mother and daughter centrioles (grey arrows), cilia with only a mother centriole (orange arrows) and cilia lacking centrioles (blue arrows). (C) Cilia with associated ciliary-associated dense vesicles (CaDV; purple), ciliary-associated vesicles (CaV; magenta) and no ciliary-associated vesicles (No CaV; cyan). Scale bars: 5 μm (B) and 10 μm (A,C).

**Which part of this research project was the most rewarding?**

Spotting the Kupffer's vesicle (KV) within the entire zebrafish embryo without a marker was a major triumph. This was particularly difficult because of the KV's tiny 40.7 µm (z-axis) size, which is minuscule compared to the entire zebrafish embryo – around 0.7 mm in diameter at 12 h post-fertilization. Another deeply satisfying achievement was the painstaking, but ultimately successful, manual segmentation of each cilium, centrosome, nucleus, lumen and KV across all 406 slices. What made these triumphs even more significant was realizing they were part of a truly collaborative journey across campuses. Our sample had quite the itinerary, travelling from Syracuse to Kedar Narayan's group at National Institutes of Health (NIH), then moving on to Jesse Aaron at Janelia Research Campus and, eventually, making its way back to NIH. Every time I think about it, it highlights to me how powerful collaboration truly is. Completing the first draft of this paper was also incredibly rewarding for me. Knowing that we, through our perseverance and the strength of our multi-institutional partnership, finally brought these data to fruition and into a coherent narrative, was a gratifying experience.

**What do you enjoy most about being an early-career researcher?**

I love the expanded opportunities to master new techniques and refine critical thinking and problem-solving abilities.

… fear often points to areas of potential growth

**What piece of advice would you give to the next generation of researchers?**

Embrace new challenges even when you feel afraid; fear often points to areas of potential growth. Each new task, be it a project or public speaking, despite initial trepidation, builds resilience and competence. It is through confronting your fears you unlock your full potential, gather unique experiences, and cultivate a mindset geared towards continuous learning and achievement.

**What's next for you?**

I am currently a postdoc, and my immediate next step is to leverage the skills acquired during my PhD to intensively focus on generating impactful publications that will significantly shape my scientific trajectory. This research goal is coupled with a strong interest in advancing global biological science education within underserved international communities. I am actively seeking opportunities to help equip laboratories in these regions, aiming to transform theoretical and abstract learning into practical, hands-on scientific engagement. We have already visited some of these laboratories, and I am excited about the steps we are taking towards fostering scientific capacity and knowledge exchange globally.
